# Transgenic expression of plastidic glutamine synthetase increases nitrogen uptake and yield in wheat

**DOI:** 10.1111/pbi.12921

**Published:** 2018-04-24

**Authors:** Mengyun Hu, Xueqiang Zhao, Qian Liu, Xia Hong, Wei Zhang, Yingjun Zhang, Lijing Sun, Hui Li, Yiping Tong

**Affiliations:** ^1^ Hebei Laboratory of Crop Genetics and Breeding Hebei Academy of Agriculture and Forestry Sciences Institute for Cereal and Oil Crops Shijiazhuang China; ^2^ State Key Laboratory for Plant Cell and Chromosome Engineering Chinese Academy of Sciences Institute of Genetics and Developmental Biology Beijing China

**Keywords:** wheat, glutamine synthetase, nitrogen use efficiency

## Abstract

The plastidic glutamine synthetase isoform (GS2) plays a key role in nitrogen (N) assimilation. We introduced *TaGS2‐2Abpro::TaGS2‐2Ab,* the favourable allele of *TaGS2‐2A* in the winter wheat (*Triticum aestivum*) variety Ji5265. Transgenic expression of *TaGS2‐2Ab* significantly increased GS2 abundance and GS activity in leaves. Two consecutive field experiments showed the transgenic lines had higher grain yield, spike number, grain number per spike and 1000‐grain weight than did the wild type under both low N and high N conditions. Analysis of N use‐related traits showed that transgenic expression of *TaGS2‐2Ab* increased root ability to acquire N, N uptake before and after flowering, remobilization of N to grains and N harvest index. Measurement of chlorophyll content and net photosynthesis rate in flag leaves during grain filling stage revealed that the transgenic lines had prolonged leaf functional duration as compared with the wild type. These results suggest that *TaGS2* plays important role in N use, and the favourable allele *TaGS2‐2Ab* is valuable in engineering wheat with improved N use efficiency and grain yield.

## Introduction

Nitrogen (N) fertilizer is indispensable for growing the world's food. Now the world's food production is facing the challenge of excess N input and low N use efficiency (NUE). An estimation of the amount of excess nutrient application in 17 major crops has revealed that ~60% of N inputs are in excess (West *et al*., 2014). It is estimated that only 30%–50% of applied N is taken up by crops, and the unused N is lost to the environment which cause soil, water and air pollution (Han *et al*., 2015; Hirel *et al*., 2011; Robertson and Vitousek, 2009; West *et al*., 2014). Therefore, breeding crops with improved NUE represents effective approach in increasing crop productivity with less N Fertilizer input (Han *et al*., 2015; Hirel *et al*., 2011).

Conventional breeding and molecular genetics have been employed to improve NUE in crops (Hirel *et al*., 2011). As NUE is governed by complicated gene networks that mediate the uptake, assimilation, remobilization and storage of N (Good *et al*., 2004; Masclaux‐Daubresse *et al*., 2010; McAllister *et al*., 2012; Xu *et al*., 2012), much more works of molecular breeding and genetic engineering approaches are required to improve NUE. Currently, many candidate genes have been identified for improving NUE in crop plants, and these candidate genes are existed in pathways relating to uptake, assimilation, remobilization and storage of N (Masclaux‐Daubresse *et al*., 2010; McAllister *et al*., 2012; Thomsen *et al*., 2014). For example, selection of natural variation or overexpression of nitrate transporter has been shown to increase grain yield and NUE in rice (*Oryza sativa*) (Fan *et al*., 2016; Hu *et al*., 2015). Manipulating N assimilation also has been shown great potential in improving NUE. A well‐known example is that transgenic expression of an alanine aminotransferase *AlaAT* using a root epidermal cell promoter in oilseed rape (*Brassica napus*) and rice increased yield and biomass under N‐limiting conditions compared with control plants (Good *et al*., 2007; Shrawat *et al*., 2008). The plant‐specific DNA BINDING WITH ONE FINGER (Dof) transcription factor *Dof1* from maize (*Zea mays*) has been shown to improve N assimilation and growth of rice under low N conditions by regulating genes involved in the tricarboxylic acid cycle (TCA cycle) and increasing carbon flow towards N assimilation (Kurai *et al*., 2011).

Once N has entered the plant, it can be assimilated into amino acids and other important nitrogenous compounds. The first step in which inorganic N is assimilated into organic composition is catalysed by glutamine synthetase (GS) (Miflin and Habash, 2002). Based on the subcellular location, GS is classified into cytosolic (GS1) and chloroplastic (GS2) isoforms. GS1 is usually encoded by a multigene family, and GS2 is often encoded by a single gene (Chardon *et al*., 2012; Miflin and Habash, 2002). We identified three *GS1* genes and one *GS2* gene in each subgenome of wheat by analysing the reference sequence of the bread wheat variety Chinese Spring (Figure S1). There is accumulating evidence supporting that both GS1 and GS2 play essential roles in efficient N use and high yield potential in major crops including wheat, rice and maize (Chardon *et al*., 2012; Hirel *et al*., 2011; Thomsen *et al*., 2014). And both of these two enzymes have been used to engineering N use efficient crops. For example, overexpressing a *GS1* gene from bean with rubisco small subunit (rbcS) promoter from rice exhibited higher root dry weight, grain weight and grain N accumulation than the nontransgenic controls in wheat (Habash *et al*., 2001). Overexpression of the GS1 gene *Gln1* with an ubiquitin promoter led to increased harvest index, N harvest index and spikelet number in rice (Brauer *et al*., 2011). In maize, overexpressing the GS1 gene *Gln1‐3* using a cassava vein mosaic virus (CsVMV) promoter increased grain number and yield (Martin *et al*., 2006). In sorghum (*Sorghum bicolor*), enhanced biomass production was observed by the overexpression of the *GS1* gene *Gln1* under low N conditions (Urriola and Rathore, 2015). However, a recent review revealed that the outcome of overexpressing *GS1* has generally been inconsistent, many studies have shown decreases in N use, biomass and yield by overexpressing *GS1* (Thomsen *et al*., 2014). One possible reason underlying the inconsistent outcome is that overexpressing *GS1* may cause metabolic imbalances, thus more refined overexpression strategies are needed to overcome metabolic bottlenecks (Thomsen *et al*., 2014). In contrast to the numerous studies on the transgenic modification of *GS1* (Thomsen *et al*., 2014), only very few studies were carried out on the effects of transgenic modifying *GS2*. Overexpressing *GS2* derived by a leaf‐specific promoter rbcS has been found to increase the growth of tobacco seedlings (Migge *et al*., 2000), and overexpressing *GS2* can enhance photorespiration and tolerance to salt stress in rice (Hoshida *et al*., 2000). To the best of our knowledge, the influence of overexpressing *GS2* on N use and grain productivity has not been reported yet.

In wheat, GS activity is proposed to be one of the best physiological markers that allow the depiction of the plant N status (Kichey *et al*., 2006, 2007), as leaf GS activity was found to positively correlate with leaf soluble protein and N content (Habash *et al*., 2007; Kichey *et al*., 2006, 2007), and grain yield (Kichey *et al*., 2007), but negatively correlate with leaf senescence (Kichey *et al*., 2007). As mentioned above, total GS activity is catalysed by GS1 and GS2. GS2 was found to be the major isozyme in the leaf tissues during vegetative growth in wheat (Bernard *et al*., 2008; Habash *et al*., 2001). When leaves aged during grain filling, there was a decrease in total GS activity (Habash *et al*., 2001; Kichey *et al*., 2007). This was mainly due to loss of GS2 and thus GS1 became a greater contributor to total GS activity (Habash *et al*., 2001). These dynamic changes in leaf GS1 and GS2 during grain filling made us to think the possibility of engineering *GS2* expression in increasing NUE and grain yield in wheat. Our previous study has identified two (a and b), six (a to f) and two (a and b) haplotypes for *TaGS2‐2A* (former name *TaGS2‐A1*), *TaGS2‐2B* (former name *TaGS2‐B1*) and *TaGS2‐2D* (former name *TaGS2‐D1*) in the mini core collection of Chinese wheat varieties, respectively. Association analysis revealed that the *TaGS2‐2Ab* haplotype showed significant association with higher shoot and root dry weigh at seedling stage, and higher thousand grain weight and higher grain N concentration (Li *et al*., 2011). Here, we found that *TaGS2‐2Ab* encoded an enzyme with higher GS activity than other haplotypes did. Field experiments showed that transgenic expression of *TaGS2‐2Ab* driven by its own promoter increased the growth and nitrate influx rate of the roots, postanthesis N uptake, leaf functional duration, NHI, grain N concentration and grain yield under both low and high N conditions.

## Results

### *In vitro* assay of TaGS2 activity

**Figure 1:**
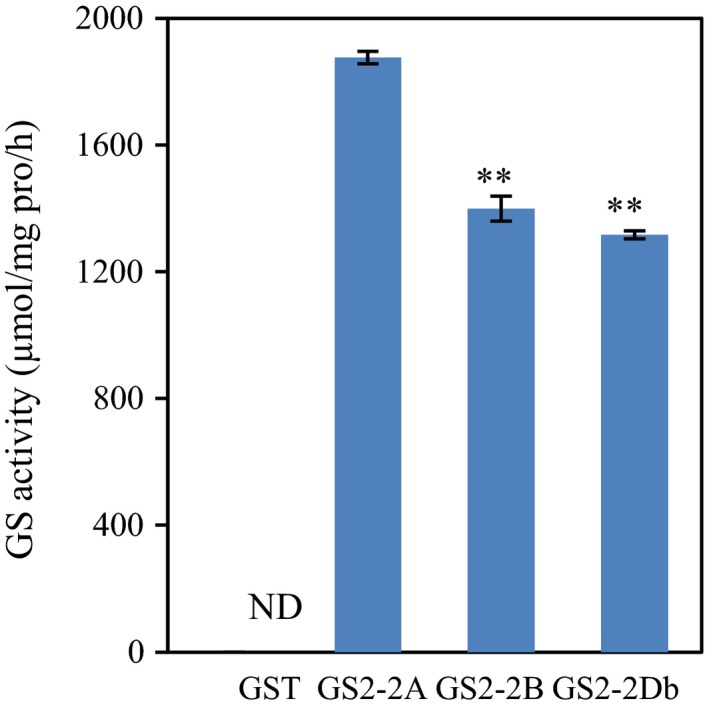
*In vitro* assay of TaGS2 activity*. GST* and N‐terminal *GST*‐tagged *TaGS2*‐*2A*, ‐*2B* and ‐2*Db* were overexpressed in *Escherichia coli*. The expressed proteins were purified to homogeneity. *In vitro* GS assays were performed as described under ‘Materials and methods'. Data represent means ± SE of four replicates. Asterisks indicate that the difference between the means of the TaGS2 activities was significant at the *P *<* *0.01 (**) level.

Our previous study has shown that there were three *TaGS2* genes located on the homologous group 2 chromosomes and have identified two (*a* and *b*), six (*a* to *f*) and two (*a* and *b*) haplotypes for *TaGS2‐2A*,* TaGS2‐2B* and *TaGS2‐2D*, respectively (Li *et al*., 2011). The ten haplotypes of *TaGS2* genes encode three kinds of putative GS2 proteins (Figure S1), TaGS2‐2A representing the sequences of TaGS2‐2Aa, 2Ab and 2Da, TaGS2‐2B representing the sequences of all TaGS2‐2B haplotypes, and TaGS2‐2Db representing the sequence of TaGS2‐2Db. Prokaryotic expression experiment *in vitro* showed that TaGS2‐2A had 40% higher GS activity than did TaGS2‐2B and TaGS2‐2Db (Figure 1).

### Selection of *TaGS2‐2Ab* transgenic lines

**Figure 2:**
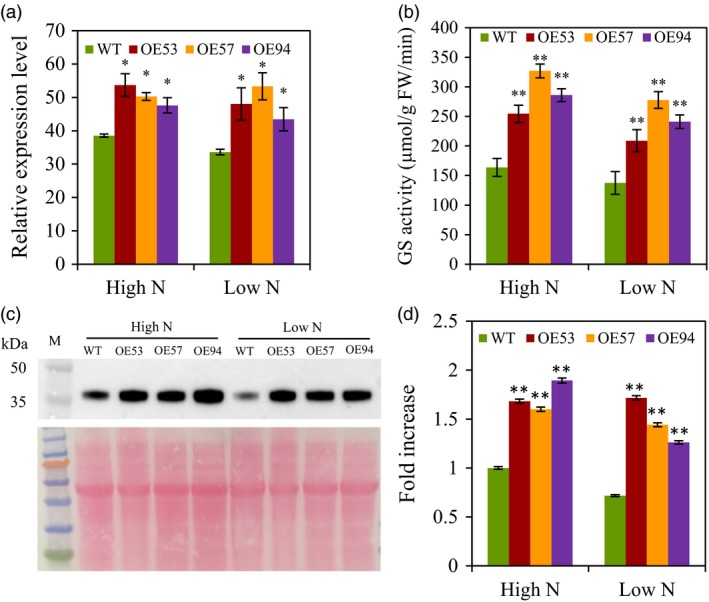
Molecular characterization of the *TaGS2‐2Ab* transgenic lines and wild control Ji5265. The transgenic lines (OE53, 57 and 94) and wild type (WT) were grown under low N and high N conditions in field experiment 2. The flag leaves were collected at 14 DPA for molecular characterization of GS2. (a) Relative expression level of total *TaGS2*. The relative expression levels were normalized to the expression of *TaACTIN*. (b) Total GS activity. (c) Protein abundance of TaGS2. Ten micrograms total chloroplastid protein was loaded in each lane. Upper panel shows the GS2 immunoblot and lower panel the Ponceau‐stained blot. (d) Quantified intensity of the TaGS2 Western bands present in (c). Data represent means ± SE of at least three replicates. Asterisks indicate that the difference between the means of the transgenic lines and WT was significant at the *P *<* *0.05 (*) and *P *<* *0.01 (**) level.

We totally obtained 85 transgenic lines by introducing *TaGS2‐2Abpro::TaGS2‐2Ab* into the wheat variety Ji2565. Thirty‐seven lines of T3 generation were further selected to evaluate the yield performance under high N conditions in 2011–2012 growing season. The results showed that the grain yield increased first with the GS activity of the flag leaves at 14 DPA, and then declined when the GS activity increased further (Figure S2). Based on the grain yield and GS activity, the three transgenic lines OE53, OE57 and OE94 were selected for further study. In field experiment 2 in 2013–2014 growing season, the transgenic lines had greener canopy than the wild type under both low N and high N conditions (Figure S3). The total *TaGS2* mRNA levels and GS activities in the flag leave at 14 DPA were investigated. All the transgenic lines had significantly higher expression *TaGS2* (Figures 2a, S4), GS activity (Figure 2b) and GS2 protein abundance (Figure 2c,d) than did the wild type under both low N and high N conditions.

### Grain yield and yield components

**Figure 3:**
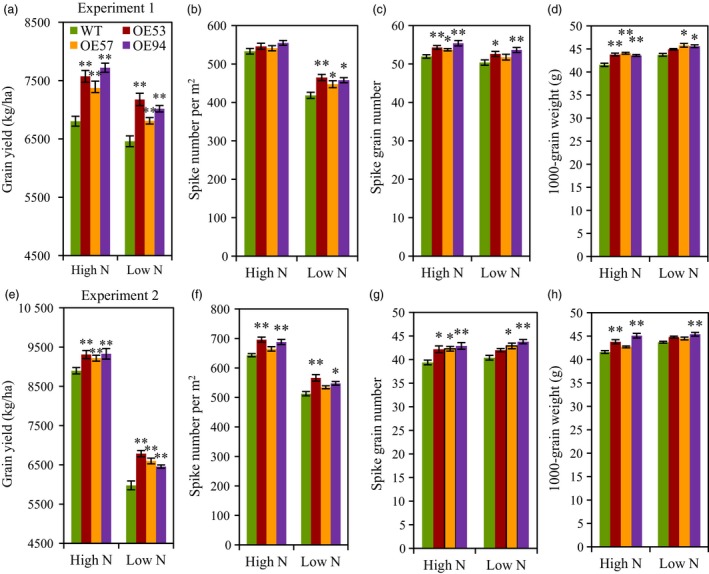
Agronomic traits of the wild type and *TaGS2‐2Ab* transgenic in the field experiments. (a–d) Grain yield per ha (a), spike number per m^2^ (b), grain number per spike (c) and 1000‐grain weight (d) in experiment 1 in 2012–2013 growing season. (e–h) Grain yield per ha (e), spike number per m^2^ (f), grain number per spike (g) and 1000‐grain weight (h) in experiment 2 in 2013–2014 growing season. Data represent means ± SE of four replicates. Asterisks indicate that the difference between the means of the transgenic lines and wild type was significant at the *P *<* *0.05 (*) and *P *<* *0.01 (**) level.

The agronomic traits of the transgenic lines and wild type were evaluated under low N and high N conditions in two consecutive field experiments. Both experiments observed significant higher grain yield in the transgenic lines than in the wild type under both N conditions (Figure 3a,e). In experiment 1 in 2012–2013 growing season, the transgenic lines had 5.4%–11.1% and 8.4%–13.5% higher grain yield than did the wild type under low N and high N conditions, respectively (Figure 3a). In experiment 2 in 2013–2014 growing season, the transgenic lines had 8.0%–13.5% and 3.6%–4.9% higher grain yield than did the wild type under low N and high N conditions, respectively (Figure 3e). We further analysed the yield components, the result showed the three transgenic lines had higher spike number, grain number per spike and 1000‐grain weight (TGW) under both low N and high N conditions, but the increasing levels depended on the transgenic line, N treatment and growing season (Figure 3b–d,f–h). We also generated transgenic lines with *TaGS2‐2Abpro::TaGS2‐2Ab* in another wheat variety Kenong199 and the results showed that the grain yield is higher in transgenic lines than that in the wild type and azygous control line under both high N and low N conditions (Figure S5).

### N use‐related traits

**Figure 4:**
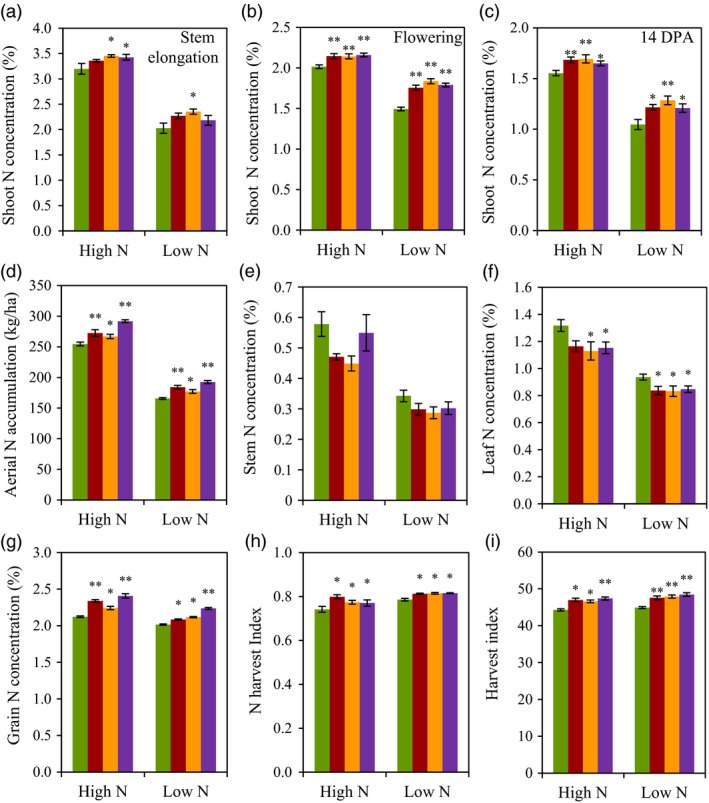
N use‐related traits of the wild type and the transgenic lines in field experiment 2 in 2013–2014 growing season. (a–c) Shoot N concentration at stem elongation (a), flowering (b) and 14 DPA (c). (d–i) Aerial N accumulation (d), stem N concentration (e), leaf N concentration (f), grain N concentration (g), nitrogen harvest index (h) and Harvest index (i) at maturity. Data represent means ± SE of four replicates. Asterisks indicate that the difference between the means of the transgenic lines and wild type was significant at the *P *<* *0.05 (*) and *P *<* *0.01 (**) level.

We investigated N use‐related traits in experiment 2. Under high N conditions, the N concentration in whole aerial parts of the three transgenic lines was 5.9%–15.4% higher at stem elongation stage (Figure 4a), 6.9%–7.1% higher at anthesis stage (Figure 4b) and 6.6%–9.2% higher at 14 days postanthesis (DPA, Figure 4c) than did the wild type. Under low N conditions, the N concentration in whole aerial parts of the three transgenic lines was 5.7%–11.8% higher at stem elongation stage (Figure 4a), 10.5%–16.3% higher at anthesis stage (Figure 4b) and 14.8%–23.2% higher at 14 DPA (Figure 4c) than did the wild type. At maturity stage, the transgenic lines had 4.8%–14.6% higher and 6.9%–16.2% higher aerial N accumulation than did the wild type under high N and low N conditions, respectively (Figure 4d). A pot experiment by ^15^N labelling fertilizer at stem elongation and flowering stage showed that the transgenic lines, OE53 and OE94, absorbed more N than did the wild type before and after flowering (Table S1). The field experiment also found that transgenic expression of *TaGS2‐2Ab* altered N distribution in aerial parts by allocating more N in grains, as the transgenic lines displayed lower leaf and stem N concentration (Figure 4e,f), higher grain N concentration (Figure 4g), N harvest index (NHI, Figure 4h) and harvest index (HI, Figure 4i) than did the wild type under both N conditions. In line with the results of grain N concentration, the transgenic lines had significantly higher concentrations of the total amino acids in grains than did the wild type (Figure S6, Table S2).

### Wheat root morphology, nitrate flux rate and expression of nitrate transporters

**Figure 5:**
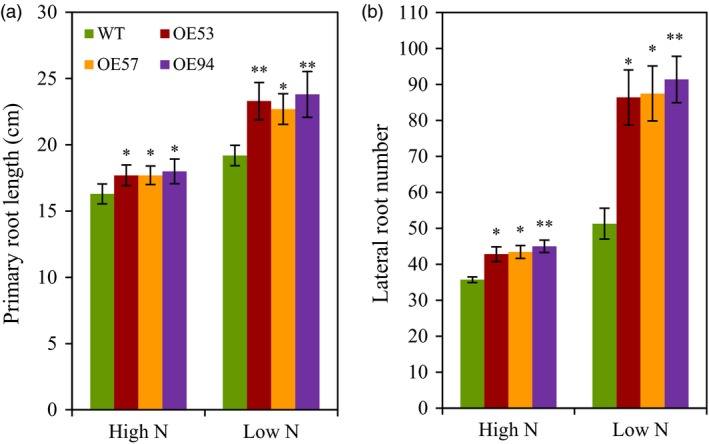
Root morphological parameters of the wild type wheat (J5265) and the transgenic lines. Wheat seedlings (7 days after germination) were grown for 12 days in a nutrient solution that contained 2 mm nitrate (high N) or 0.2 mm nitrate (low N). (a) Primary root length. (b) Lateral root number. Data represent means ± SE of at least five replicates. Asterisks indicate that the difference between the means of the transgenic lines and wild type was significant at the *P *<* *0.05 (*) and *P *<* *0.01 (**) level.

**Figure 6:**
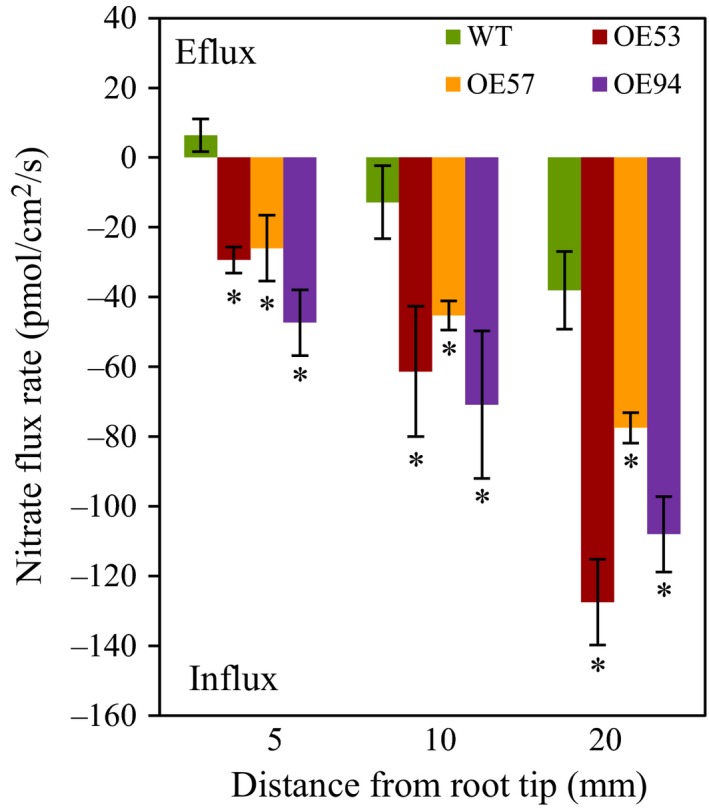
Nitrate flux rate. NO_3_^−^ flux rate was measured by SIET with 0.2 mm NO_3_^−^ in the measuring solution. Seedlings were pretreated with 0.2 mm nitrate and were then used to measure nitrate influx rate at the root tip surface in measuring solutions that contained 0.2 mm nitrate. Positive and negative flux values indicate efflux and influx, respectively. Data represent means ± SE of at least three replicates. Asterisks indicate that difference between the wild type and the transgenic lines was significant at the *P *<* *0.05 level.

To understand the mechanisms by which *TaGS2‐2Abpro::TaGS2‐2Ab* increased N uptake, we first investigated how *TaGS2‐2Ab* affected root growth. In a hydroponic culture system with plants at the seedling stage, we observed that the three transgenic lines had longer primary root length and more lateral root number than did the wild type (Figure 5). We next measured nitrate flux rates of roots. The transgenic lines displayed significantly higher nitrate influx rates at root surfaces 5, 10 and 20 mm from the root tip than that did the wild type when the nitrate flux rate was recorded in a measuring solution containing 0.2 mm nitrate (Figure 6). We also analysed the expression of nitrate transporter genes. In roots, the three transgenic lines had higher *TaNRT2.1* and *TaNPF6.3* expression than did the wild type (Figure S7). These results suggest that the overexpression of *TaGS2‐2Abpro::TaGS2‐2Ab* up‐regulated the expression of genes involved in nitrate transport. These results also indicate that overexpression of *TaGS2‐2Abpro::TaGS2‐2Ab* in wheat enhanced nitrate uptake rate, possibly by up‐regulating the expression of nitrate transporters.

### N concentration, chlorophyll concentration and net photosynthesis rate in flag leaves

**Figure 7:**
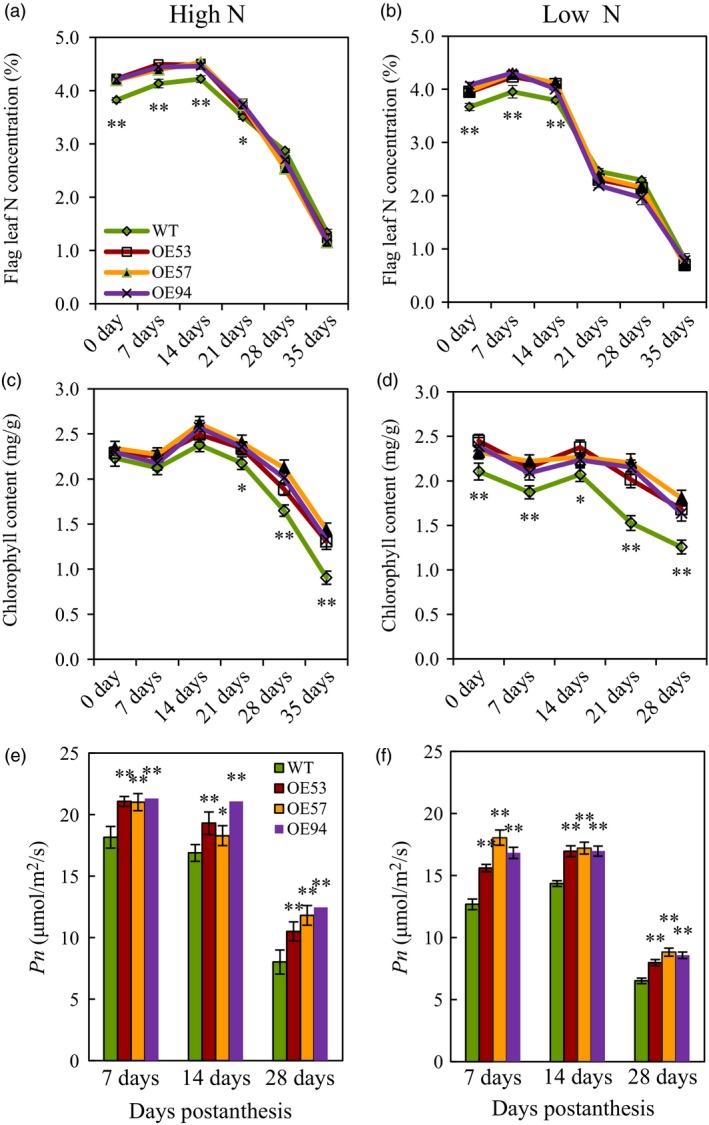
N content, chlorophyll content and net photosynthesis rate in flag leaves of the transgenic lines and wild type in experiment 2 in 2013–2014 growing season. (a and b) Flag leaf N content under high N (a) and low N (b) conditions. (c and d) Chlorophyll content in flag leaves under high N (c) and low N (d) conditions. (e and f) Net photosynthesis rate (*Pn*) under high N (e) and low N (f) conditions. Data represent the mean ± SE of 4 replications. Asterisks indicate that the difference between the means of the transgenic lines and wild type was significant at the *P *<* *0.05 (*) and *P *<* *0.01 (**) level.

During grain filling, the flag leaves of the transgenic lines had higher N concentration before 21 DPA under high N condition (Figure 7a) and before 14 DPA under low N conditions (Figure 7b), higher chlorophyll concentration after 21 DPA under high N conditions (Figure 7c) and at all the measuring dates under low N conditions (Figure 7d), and higher *Pn* at all the measuring dates under both N conditions (Figure 7e,f) than those of wild type. We also found that the transgenic lines had larger flag leaves (Figure S8), higher soluble proteins at 14 DPA (Figure S9), and higher superoxide dismutase (SOD) activity (Figure S10a,b), lower malondialdehyde (MDA) concentration (Figure S10c,d) in the flag leaves at 7 DPA–35 DPA than did the wild type under both N conditions. These results indicated that the transgenic lines had increased flag leaf size and prolonged flag leaf functional duration.

## Discussion

### Transgenic expression of *TaGS2‐2Ab* leads to an N efficient wheat ideotype with high grain mass, large harvest index and N harvest index, and high grain N concentration

Manipulating N assimilation has been proposed an important strategy to improve NUE. GS, which catalyses the first step in assimilating inorganic N into organic composition (Miflin and Habash, 2002), has been used to improve NUE in many crops including wheat (Chardon *et al*., 2012; Hirel *et al*., 2011; Thomsen *et al*., 2014). However, the majority of the studies were conducted in greenhouse to evaluate the phenotypes of the transgenic lines. And the effects of overexpressing *GS* on N use‐related physiological traits, biomass and yield have generally been inconsistent (Thomsen *et al*., 2014). The two consecutive field experiments in present study showed that transgenic expression of *TaGS2‐2Ab* increased flag leaf GS activity, leaf functional duration, N uptake, grain N concentration, NHI, HI, grain yield and yield components under both low N and high N conditions. These merits of the transgenic lines meet the requirement for an N efficient wheat ideotype with high grain mass, large HI and NHI, and high GPC (Chardon *et al*., 2012). Two strategies might be important for the successful use of *TaGS2‐2Ab* in engineering N efficient wheat. The first one was to optimize leaf GS activity. The correlation between flag leaf GS activity and grain yield of T3 transgenic lines showed that extremely high leaf GS activity had negative effect on grain yield (Figure S2). We observed that the transgenic lines with very high leaf GS activity exhibited excessive stay‐green and had reduced TGW. The second one was to drive the expression of *TaGS2‐2Ab* by its own promoter. Overexpression of *GS* may cause metabolic imbalances and inhibit N use and plant growth, thus more refined overexpression strategies combined with tissue and cell specific targeting are needed to overcome metabolic bottlenecks in engineering N efficient crops (Thomsen *et al*., 2014). Optimizing GS activity and driving *TaGS2‐2Ab* by its own promoter may avoid the possible metabolic imbalances.

### Transgenic expression of *TaGS2‐2Ab* increases N uptake and remobilization

The size and architecture of root system is an important variable for ensuring adequate access to N in plant (Miller and Cramer, 2005). The increased primary root length and lateral root number (Figure 5) combined with the increased nitrate influx rates (Figure 6) provided a strong basis for the *TaGS2‐2Ab*‐transgenic wheat lines to efficiently acquire soil N resources, as has been shown in the field experiment 2 (Figure 4d) and in the pot experiment (Table S1). We found that transgenic expressing *TaGS2‐2Ab* increased expression of *TaNRT2.1* and *TaNPF6.3* (Figure S7). NRT2 function as high‐affinity nitrate transporter. Increasing expression of *OsNRT2.1* through transgenic approach enchanted N uptake before and after heading in rice (Chen *et al*., 2016). In wheat, it has been reported that root expression of transporter *TaNRT2.1* is related with postanthesis N uptake (Taulemesse *et al*., 2015). TaNPF6.3 is orthologous to *Arabidopsis* AtNPF6.3/NRT1.1 which functions as dual‐affinity nitrate transporter (Wang *et al*., 1998). Overexpression of *OsNPF6.3*/*NRT1.1b* increased N uptake and grain yield in rice (Hu *et al*., 2015). As such, the increased expression of *TaNRT2.1* and *TaNPF6.3* may partially explain the enhanced nitrate influx (Figure 6), and N uptake before and after flowering (Table S1) by transgenic expressing *TaGS2‐2Ab*.

Grain yield and GPC are two major targets of wheat breeding. However, the strong genetic negative correlation between grain yield and GPC in wheat makes it difficult to improve these two traits simultaneously (Bogard *et al*., 2010). Our current study showed transgenic expression of *TaGS2‐2Ab* increased not only grain yield but also grain N concentration under both low N and high N condition, enabling *TaGS2‐2Ab* valuable in shifting the negative correlation between grain yield and GPC. Usually, increased grain N concentration can be accomplished through two ways, one is to increase N uptake, and another one is to improve partitioning of N to the grain through remobilization. Transgenic expressing *TaGS2‐2Ab* increased N uptake before anthesis and more apparently after anthesis (Table S1). It has been proposed that increasing postanthesis N uptake is important to increase GPC while maintaining high grain productivity (Bogard *et al*., 2010; Monaghan *et al*., 2001). Several lines of evidence supported that transgenic expressing *TaGS2‐2Ab* increased N remobilization. Firstly, the transgenic lines had higher shoot N concentrations at flowering (Figure 4b), but lower leaf and stem N concentrations than did the wild type at maturity (Figure 4e,f). Secondly, the N concentrations in flag leaves of the transgenic lines were higher than those of wild type at early stage of grain filling, but were similar with those of wild type at later stage of grain filling (Figure 7a,b). Finally, the transgenic lines had higher NHI than did the wild type (Figure 4h). GS1 and GS2 isoforms play nonoverlapping roles in plant N assimilation. During leaf senescence, GS1 was the predominant isoform of GS enzymes and was thought to play major roles in assimilating ammonia during the critical phases of remobilization of N to the grain in wheat (Bernard *et al*., 2008) while GS2 was proposed a major role in ammonium assimilation within photorespiratory N cycling (Bernard and Habash, 2009). Our data clearly showed that GS2 was also important in remobilization of N to the grain. However, the underlying mechanisms are needed to explore in future research.

### Transgenic expression of *TaGS2‐2Ab* prolongs leaf functional duration

Leaf senescence is the final stage of leaf development in nature which is an important process for crop yield. Delaying leaf senescence and extending the duration of leaf photosynthesis during grain filling is a possible route for increasing grain yields (Gaju *et al*., 2011; Hebbar *et al*., 2014; Kichey *et al*., 2007; Richards, 2000). Our current study found that transgenic expressing *TaGS2‐2Ab* prolonged leaf functional duration (Figure 7c–f), providing strong leaf metabolic basis for the increased grain yield and TGW. Although the transgenic lines had higher chlorophyll content after 21 DPA (Figure 7c,d) and higher *Pn* at 28 DPA (Figure 7e,f) than did the wild type, they had similar leaf N concentrations with the wild type after 21 DPA (Figure 7a,b). This phenomenon implied that the transgenic lines had higher photosynthetic capacity using similar amount of N. Although the underlying mechanisms are worthy to be elucidated, the increased SOD activity and reduced MDA content (Figure S10) by expressing *TaGS2‐2Ab* might gave partial explanation. In north China (where our field experiments located), during the late grain filling stage, wheat often experiences hot weather combined with high sun light, which may cause photo‐oxidative damage to photosynthetic apparatus (Yang *et al*., 2006). The increased SOD activity and reduced MDA content suggested that the transgenic lines had a more efficient antioxidant system and higher capacity for the scavenging of reactive oxygen species favourable for resistance to photo‐oxidative stress. It has been shown that during leaf senescence, GS2 experienced rapid loss and GS1 became the major GS isoform (Habash *et al*., 2001). The prolonged leaf functional duration by transgenic expressing *TaGS2‐2Ab* suggested that a low GS2 activity may be a limiting factor for leaf function during the late grain filling stage.

In summary, *TaGS2‐2Ab* is a valuable gene resource for breeding wheat with grain yield and GPC under a range of soil N conditions. Besides transgenic modification, marker‐assisted selection of different *TaGS2* haplotypes may be effective in improving NUE and yield, as our previous study has already shown that the favourable haplotypes of *TaGS2* were associated with N use‐ and yield‐related traits (Li *et al*., 2011). Although we observed that transgenic expression of *TaGS2‐2Ab* increased root ability to acquire N and N remobilization, further studies are needed to dissect the underlying mechanisms.

## Materials and methods

### Plant materials

The wheat (*Triticum aestivum*) varieties Xiaoyan 54, Zhengmai 9023 and Ji5265 were used in this study. Xiaoyan 54 was used to isolate the sequences of the *TaGS2‐2Ab* gene, and Zhengmai 9023 was used to isolate the cDNA sequences of *TaGS2‐2A*,* TaGS2‐2B* and *TaGS2‐2Db*. Ji5265 was used to generate the transgenic lines. Ji5265 is a winter wheat variety which was commercially released in 2007.

### *In vitro* assay of TaGS2 activity

We used the total cDNA from wheat variety Zhengmai 9023 as the template and amplified the ORF region for *TaGS2‐2A*,* TaGS2‐2B* and *TaGS2‐2Db* cDNA by PCR with the universal primers of *TaGS2*. The primers used were forward (5′‐CGGGATCCATGGCGCAGGCGGTGGT‐3′) and reverse (5′‐AAGGAAAAAAGCGGCCGCTCATACCTTCAGCGCCAGCTTC‐3′). These amplified ORFs were inserted into pGEX4T‐1 plasmid at *BamH*I and *Not*I sites. All the constructs of three *TaGS2* cDNA were confirmed by sequencing the ligation products of pGEX4T‐1 plasmids. The constructs, pGEX4T‐1‐TaGS2‐2A, pGEX4T‐1‐TaGS2‐2B, pGEX4T‐1‐TaGS2‐2Db and empty pGEX4T‐1 vector, were transformed into BL21 (DE3) plysS cells (TransGen Biotech Inc., Beijing, China). Protein productions were induced by the addition of isopropyl‐β‐d‐thiogalactoside to a final concentration of 0.05 mm and incubated in shaker with 150 rpm overnight at 20 °C. After induction, cells were harvested by centrifugation at 6000 ***g*** for 30 min at 4 °C. After decanting the supernatant, the resulting pellet was suspended in phosphate buffer solution (PBS, pH 7.4) and sonicated with three 5‐s bursts using an ultrasonic homogenizer (Scientz‐IID; Ningbo Scientz Biotechnology Co. Ltd., Ningbo, China). The lysate was centrifuged at 1 4000 ***g*** for 30 min at 4 °C, and the supernatants were transferred to an equilibrated for purification. GST‐TaGS2‐2A, GST‐TaGS2‐2B and GST‐TaGS2‐2Db fusion proteins were produced and purified to homogeneity as described previously (Huang *et al*. 2007). In brief, crude protein extracts were loaded onto a glutathione sepharose 4B column (GE Healthcare, Waukesha, WI), and then, after washing seven times with PBS buffer, GST‐TaGS2‐2A, GST‐TaGS2‐2B and GST‐TaGS2‐2Db fusion proteins were eluted using 5 mm reduced glutathione in 50 mm Tris–HCl, pH 8.0 and then concentrated by Amicon Ultra‐15 Centrifugal Filter Units (Millipore, Darmstadt, Germany). The GS activities of the purified proteins were determined using Glutamine Synthetase Detection Kit A047 (Nanjing Jiancheng Biotechnology, Nanjing, China).

### Plasmid construction and wheat transformation

The complete open reading fragment (ORF) of *TaGS2‐2Ab* (GQ169685.1) was amplified from the total cDNA prepared from wheat variety Xiaoyan 54 using an reverse transcription (RT)‐PCR system (TaKaRa, Dalian, China) in accordance with the manufacturer's instructions. The primers used were forward (5′‐ATGGCGCAGGCGGTGGTGCCGGCGATG‐3′) and reverse (5′‐TCATACCTTCAGCGCCAGCTTCTTG‐3′). The promoter of *TaGS2‐2Ab* (*TaGS2‐2Abpro*) was amplified from the genomic DNA of Xiaoyan 54. The primers used were forward (5′‐TGGAGGGTGACTGCTCCAGAGTTC‐3′) and reverse (5′‐CTTCCCCTTCAGCGCTAGCTGATC‐3′). The amplified *TaGS2‐2Ab* ORF was inserted in frame between Ubiquitin promoter and 3′NOS at *BamH*I and *Kpn*I sites of the modified pACH25 vector (Christensen *et al*., 1992). Then, the Ubiquitin promoter was replaced with *TaGS2‐2Abpro* at *Pst*I and *BamH*I sites, resulting in the *TaGS2‐2Abpro::TaGS2‐2Ab‐*pACH25 construct. The construct was transformed into immature embryos of wheat variety Ji5265 using the method described by Wang *et al*. (2013).

### Field experiments

The field experiments were conducted in the Dishang Experimental Station which belongs to the Institute of Grain and Oil Crops, Hebei Academy of Agriculture and Forestry Sciences in Shijiazhuang, Hebei Province. Two consecutive field experiments were conducted in 2012–2013 (transgenic T4 generation, experiment 1) and 2013–2014 (transgenic T5 generation, experiment 2) growing seasons. Both experiments had two treatments each with four replications. The high N treatment had 22.5 g N/m^2^ in the form of urea with 13.5 g N/m^2^ applied prior to sowing and 9.0 g N/m^2^ applied at the stem elongation stage. The low N treatment had no N application in experiment 1 and 6.75 g N/m^2^ applied prior to sowing in experiment 2. Both treatments were applied 8 g/m^2^ phosphorus (P) as calcium superphosphate and 10 g/m^2^ potassium (K) as potassium sulphate. For each genotype in each replicate, the plot was 1.7 m × 8.4 m which includes eight rows and the rows were spaced 20 cm apart. The sowing density was set at 270 germinating seeds per m^2^. Ten plants in each plot were randomly collected to measure N concentration in aerial parts at stem elongation, anthesis and 14 DPA. At maturity, twenty plants in each plot were randomly collected to measure N concentration and dry weight of leaves, stems and grains, and grain number per spike, and 1000‐grain weight (TGW). Plant density and spike density were recorded on two rows each with 1 m long in each plot. Grain yield was recorded in the whole plot. Aerial N accumulation at maturity was estimated as (grain yield × grain N concentration)/NHI. Total N concentration in plant samples was measured using a semi‐automated Kjeldahl method (Tecator Kjeltec Auto 1030 Analyzer; Tecator, Hoganas, Sweden).

### Quantitative real‐time PCR

Total RNA from plant tissues was extracted with TRIzol reagent (Thermo Fisher Scientific, Waltham, MA). The first‐strand cDNA was synthesized from 2 μg of DNase I‐treated total RNA using murine leukaemia virus reverse transcriptase (Promega, Madison, WI). Quantitative real‐time RT–PCR analysis was performed with a LightCycler 480 engine (Roche, Mannheim, Germany) using the LightCycler480 SYBR Green I Master Mix (Roche). *ACTIN2* mRNA was used as an internal control. The primers used for *TaGS2*‐*2A* were TaGS2F (5′‐CCGAGACCACGATCCTGT‐3′) and TaGS2R (5‐GGTCCTTCATACCTTCAGCG‐3′). The primers used for *ACTIN2* were TaActinF (5′‐ACCTTCAGTTGCCCAGCAAT‐3′) and TaActinR (5′‐CAGAGTCGAGCACAATACCAGTTG‐3′).

### Western blot analysis

The abundant of TaGS2 protein was detected the flag leaves at 14 DPA. Twenty flag leaves were randomly collected in each plot in experiment 2. The chloroplast protein was extracted using a commercial Chloroplast Protein Isolation Kit (BestBio, Shanghai, China). Protein concentrations were determined using the BCA protein assay kit (Thermo Fisher Scientific). The extracted proteins were separated on 10% acrylamide gels (Invitrogen Life Technologies, Carlsbad, CA), and Western blot analyses were performed using a GS specific antibody raised against GS2 in rabbits (Abmart, Shanghai, China). Broad range prestained standards (GenStar Biosolutions Co. Ltd., Beijing, China) were used as markers.

### Measurement of GS activity

Twenty flag leaves were randomly collected in each plot 14DPA in experiment 2. The samples were grounded to a fine powder under liquid N and then homogenized in an extraction buffer containing 50 mm Tris‐HCl (pH 7.6), 1.0 mm EDTA, 1.0 mm MgC12, 10 mm 2‐ME, 1.0 mm DTT and 0.5% (w/v) insoluble PVP. The homogenate was centrifuged at 10 000 ***g*** for 20 min for two times at 4 °C. The supernatant fraction was used for the assay of GS activity. The GS activity was determined using Glutamine Synthetase Detection Kit A047 (Nanjing Jiancheng Biotechnology).

### Measurement of chlorophyll concentration and net photosynthesis rate

Net photosynthesis rates of ten randomly selected flag leaves in each plot were measured at 0, 7, 14, 21, 28 and 35 DPA with a LI‐COR 6400 portable photosynthesis system (LiCor Inc., Lincoln, NE). All measurements were conducted in the morning from 9:00 to 11:00 am on a clear day. For measuring chlorophyll concentration, 20 flag leaves were randomly collected. Chlorophyll concentration was measured as described by Arnon (1949).

### Root morphological analysis

The root morphological parameters of the transgenic lines (T5 generation) and wild type Ji5265 were measured. The nutrient solution and plant growth conditions were described previously (Wang *et al*., 2013). Briefly, wheat seedlings (7 days after germination) were grown for 12 days in a nutrient solution that contained 0.2 mm nitrate (low N) or 2.0 mm nitrate (high N). The length of longest primary root was measured by a ruler, and the lateral root number was accounted manually.

### Measurements of root nitrate flux rate

Wheat seedlings (7 days after germination) were grown for 7 days in a nutrient solution that contained 0.2 mm nitrate. The roots of these plants were then transferred to a measuring solution containing 0.2 mm KNO_3_, 0.1 mm CaCl_2_ and 0.3 mm MES (pH6.0), and allowed to balance for 10 min. The net nitrate fluxes were measured as described previously (He *et al*., 2015). The maximum nitrate flux rates along the root were recorded.

### Statistical analysis of data

One‐way analysis of variance was performed using SAS statistical software (SAS Institute Inc., Cary, NC).

## Author contributions

M.H. and X.Z. performed most of the experiments; Q.L. generated transgenic lines; Y.Z. participated in field experiments; L.S. identified transgenic lines; X.H. and W.Z. performed Western bolt experiment and measured enzyme activity; Y.T. and H.L. designed the experiments and analysed the data; Y.T. and X.Z. supervised and complemented the writing.

## Supporting information


**Figure S1** (a) Alignment of TaGS2 protein sequences. (b) Phylogenetic tree of glutamine synthetase and its homologues in plants.
**Figure S2** Correlation of GS activity and grain yield.
**Figure S3** Growth performance of the *TaGS2‐2Ab* transgenic lines (a) and wild control Ji5265 under low N conditions (b).
**Figure S4** Expression levels of *TaGS2* in shoots (a) and roots (b) of the transgenic lines and wild type Ji5265 grown in high N and low N.
**Figure S5** Grain yield of the wild type Kenong199 and *TaGS2‐2Ab* transgenic in the field experiments.
**Figure S6** The amino acids content in grains of the transgenic lines and wild type Ji5265.
**Figure S7** Expression levels of *TaNRT2.1* and *TaNPF6.3* in shoots and roots of of the transgenic lines and wild type Ji5265 grown in high N and low N.
**Figure S8** Flag leaf senescence in transgenic lines is delayed as compared with wild type Ji5265.
**Figure S9** Soluble protein concentrations in the flag leaves of the transgenic lines and wild type Ji5265 at 14 days postanthesis.
**Figure S10** The SOD activity and MDA concentration of the flag leaves in the wild type Ji5265 and transgenic lines during grain filling in field experiment 2.

**Table S1** Aerial N accumulation in the pot experiment.
**Table S2** The amino acids content in grains of the transgenic lines and wild type Ji5265.
