# Supplementary material for: Transgenic expression of plastidic glutamine synthetase increases nitrogen uptake and yield in wheat
Source: Plant Biotechnol J. 2018 Apr 24;16(11):1858–67. doi: 10.1111/pbi.12921 (PMC6181211; doi:10.1111/pbi.12921)
Supplement: Supplementary file 1 — Figure S1 (a) Alignment of TaGS2 protein sequences. (b) Phylogenetic tree of glutamine synthetase and its homologues in plants. Figure S2 Correlation of GS activity and grain yield. Figure S3 Growth performance of the TaGS2‐2Ab transgenic lines (a) and wild control Ji5265 under low N conditions (b). Figure S4 Expression levels of TaGS2 in shoots (a) and roots (b) of the transgenic lines and wild type Ji5265 grown in high N and low N. Figure S5 Grain yield of the wild type Kenong199 and TaGS2‐2Ab transgenic in the field experiments. Figure S6 The amino acids content in grains of the transgenic lines and wild type Ji5265. Figure S7 Expression levels of TaNRT2.1 and TaNPF6.3 in shoots and roots of of the transgenic lines and wild type Ji5265 grown in high N and low N. Figure S8 Flag leaf senescence in transgenic lines is delayed as compared with wild type Ji5265. Figure S9 Soluble protein concentrations in the flag leaves of the transgenic lines and wild type Ji5265 at 14 days postanthesis. Figure S10 The SOD activity and MDA concentration of the flag leaves in the wild type Ji5265 and transgenic lines during grain filling in field experiment 2. [file PBI-16-1858-s002.docx]

**A**

**B**


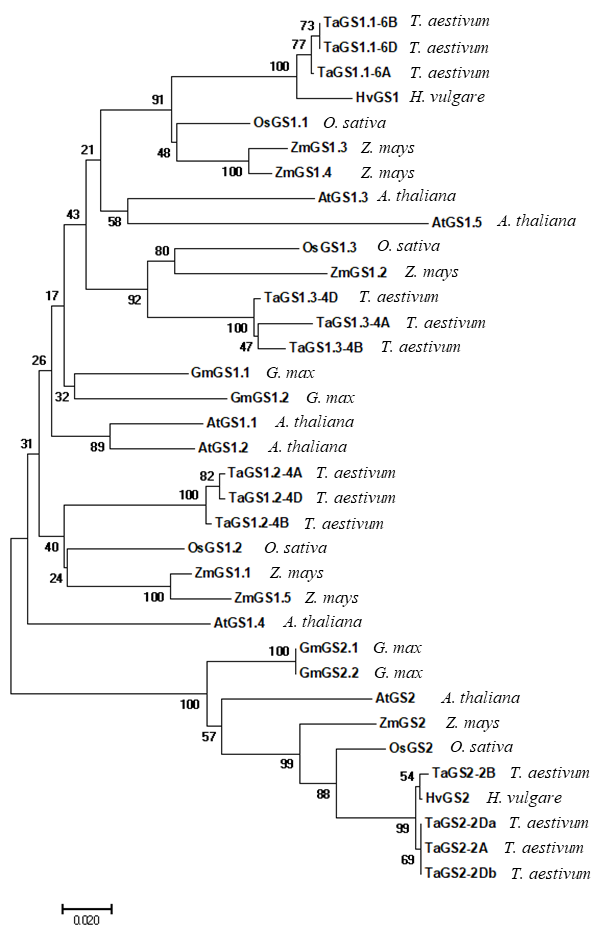


**Figure S1. (A) Alignment of TaGS2 protein sequences**. **(B) Phylogenetic tree of Glutamine synthtase and its homologues in plants.** GenBank accession number and transcript assembly number for each sequence used in the tree are listed below. *Triticum aestivum*: TaGS1.1-6A (Ta-GS1a, AAZ30057), TraesCS6A01G298100; TaGS1.1-6B (TaGS1b, AAZ30058), TraesCS6B01G327500; TaGS1.1-6D (TaGS1c, AAZ30059), TraesCS6D01G383600LC; TaGS1.2-4A, TraesCS4A01G063800; TaGS1.2-4B (Ta-GSr1, AAR84347), TraesCS4B01G240900; TaGS1.2-4D (Ta-GSr2, AAR84348), TraesCS4D01G240700; TaGS1.3-4A, TraesCS4A01G266900; TaGS1.3-4B (Ta-GSe2, AAR84350), TraesCS4B01G047400; TaGS1.3-4D (Ta-GSe1, AAR84349), TraesCS4D01G047400; TaGS2-2A, TraesCS2A01G500400; TaGS2-2B, TraesCS2B01G528300; TaGS2-2D, TraesCS2D01G500600; *Oryza sativa*: OsGS2, CAA32462; OsGS1.1, CAA32461; OsGS1.2, CAA32460; OsGS1.3, AAK18848; *Zea mays*: ZmGS2, CAA46724; ZmGS1.1, CAA46719; ZmGS1.2, CAA46720; ZmGS1.3, CAA46721; ZmGS1.4, CAA46722; ZmGS1.5, CAA46723; *Hordeum vulgare*: HvGS2, CAA37643; HvGS1, CAA48830; *Arabidopsis thaliana*: AtGS1.1 (At5g37600), NP_198576; AtGS1.2 (At1g66200), NP_176794; AtGS1.3 (At3g17820), NP_188409; AtGS1.4 (At5g16570), NP_568335; AtGS1.5 (At1g48470), NP_175280; AtGS2 (At5g35630), AAB20558; *Glycine max*; GmGS1.1(P24099); GmGS1.2(O82560.1); GmGS2.1(XP_003546121.1); GmGS2.2(XP_006597566.1). Sequences of the Glutamine Synthtase proteins were aligned with Clustal X, and the phylogenetic tree was constructed using the neighbour-joining method in MEGA7.0. The scar bar represents a distance of 0.02 changes per amino acid.

**Figure S2.** **Correlation of GS activity and grain yield. GS activity in flag leaves was measured at 14 days after flowering.**

**Figure S3**. **Growth performance of the *TaGS2-2Ab* transgenic lines (A) and wild control Ji5265 under low N conditions.**

**Figure S4.** **Expression levels of *TaGS2* in shoots (A) and roots (B) of the transgenic lines and wild type Ji5265 grown in high N and low N.**

Wheat seedlings (7 days after germination) were grown for 12 days in a nutrient solution that contained 0.2 mM nitrate (Low N) or 2.0 mM nitrate (High N). Data are means ± SE of three replicates. Asterisks indicate that the difference between the means of the transgenic lines and wild type was significant at the *P* < 0.05 (*) and *P* < 0.01 (**) level.

**Figure S5.** **Grain yield of the wild type KenongN199 and *TaGS2-2Ab* transgenic in the field experiments.**

Data represent means ± SE of four replicates. WT, Kenong199; GS2-35, GS2-54 and GS2-63, the transgenic lines; NC, the azygous control line separated from the T2 plants of the transgenic line GS2-35. Data are means ± SE of four replicates. Asterisks indicate that the difference between the means of the transgenic lines and wild type was significant at the *P* < 0.05 level.

**Figure S6. The amino acids content in grains of the transgenic lines and wild type** **Ji5265.**

The amino acid profiles for both essential and nonessential amino acid were obtained using the Biochrom 30 amino acid analyzer (Amersham, Cambridge, Britain). In a test tube, 10 mL of 6 N HCl were added to 50 mg sample. The test tube was vacuated by nitrogen, sealed, and placed in an oven at 110 °C for 24 h and then allowed to cool to room temperature. The hydrolyzate was filtered to remove the visible sediments and then evaporated to dryness under vacuum ant 60 °C. The hydrolyzate was dissolved in 1 mL buffer (pH 2.2). A known volume (20 µL) was injected into the amino acid analyzer to estimate the amino acid profile for each sample (tryptophan could not be determined by this method). Data are means ± SE of four replicates. Asterisks indicate that the difference between the means of the transgenic lines and wild type was significant at the P <0.05 (*) and P <0.01 (**) level.

**Figure S7.** **Expression levels of *TaNRT2.1* and *TaNPF6.3* in shoots and roots of of the transgenic lines and wild type Ji5265 grown in high N and low N.**

Wheat seedlings (7 days after germination) were grown for 12 days in a nutrient solution that contained 0.2 mM nitrate (Low N) or 2.0 mM nitrate (High N). The relative expression levels of *TaNRT2.1* and *TaNPF6.3* were normalized to the expression of *TaActin*. Data are means ± SE of three replicates. Asterisks indicate that the difference between the means of the transgenic lines and wild type was significant at the *P* < 0.05 (*) level.


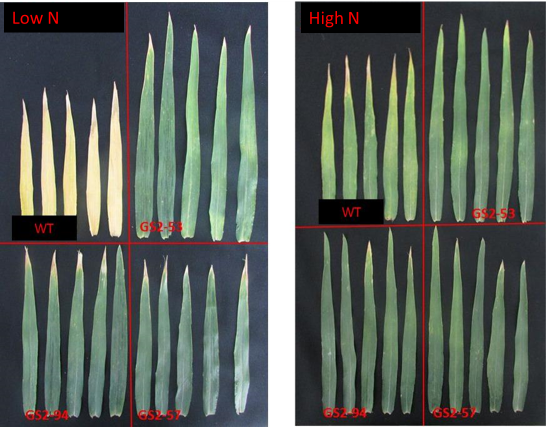


**Figure S8. Flag leaf senescence in transgenic lines is delayed as compared with wild type Ji5265.**

The leaf phenotype of the transgenic lines and wild type were investigated at 28 days after anthesis in field experiment 2.

**Figure S9**. **Soluble protein concentrations in the flag leaves of the transgenic lines and wild type Ji5265 at 14 days post-anthesis.**

For soluble protein analysis, plant materials were homogenized by grinding the freshly harvested leaves on ice with extraction buffer (10 mmol/L Trizma [pH 7.5], 5 mmol/L sodium glutamate, 10 mmol/L MgSO_4_, 1 mmol/L dithiothreitol, 10% [V/V] glycerol and 0.05% [V/V] Triton X-100). The homogenates were then centrifuged at 12000g for 20 min at 4 °C. The soluble protein concentration of the extract was quantified using a Bicinchoninic Acid Protein Assay Kit (Sigma-Aldrich, St. Louis, USA) and bovine serum albumin was used as the standard protein. Data are means ± SE of four replicates. Asterisks indicate that the difference between the means of the transgenic lines and wild type was significant at the *P* < 0.05 (*) and *P* < 0.01 (**) level.

**Figure S10. The SOD activity and MDA concentration of the flag leaves in the wild type Ji5265 and transgenic lines during grain filling in field experiment 2.**

For measuring malondialdehyde (MDA) concentration and superoxide dismutase (SOD) activity, 20 flag leaves were randomly collected. Lipid peroxidation was measured in terms of MDA concentration (Dhindsa et al., 1981). SOD activity was estimated by measuring inhibition of the reduction of nitroblue tetrazolium as described previously (Dhindsa et al., 1981). Data are means ± SE of four replicates. Asterisks indicate that the difference between the means of the transgenic lines and wild type was significant at the P <0.05 (*) and P <0.01 (**) level.

**Reference**

**Dhindsa RS, Plumbdhindsa P, Thorpe TA** (1981) Leaf senescence correlated with increased levels of membrane-permeability and lipid-peroxidation, and decreased levels of superoxide-dismutase and catalase. Journal of Experimental Botany **32:** 93-101
