# Supplementary material for: Transgenic expression of plastidic glutamine synthetase increases nitrogen uptake and yield in wheat
Source: Plant Biotechnol J. 2018 Apr 24;16(11):1858–67. doi: 10.1111/pbi.12921 (PMC6181211; doi:10.1111/pbi.12921)
Supplement: Supplementary file 2 — Table S1 Aerial N accumulation in the pot experiment. Table S2 The amino acids content in grains of the transgenic lines and wild type Ji5265. [file PBI-16-1858-s001.docx]

**Table S1. Aerial N accumulation in the pot experiment.**

| ^15^N labeling time | Lines | Total nitrogen of plant (mg) | Ndff (%) | ^15^N uptake amount (mg) | ^15^N utilization ratio (%) |
| --- | --- | --- | --- | --- | --- |
| Stem elongation | WT | 124.92±2.37bc | 25.61c | 31.99c | 5.33bc |
|  | OE53 | 144.04±4.51a | 26.25c | 38.99b | 6.72ab |
|  | OE94 | 135.20±2.53ab | 26.75c | 36.17bc | 6.03b |
| Anthesis | WT | 118.98±2.12c | 20.61d | 28.07d | 4.09c |
|  | OE53 | 146.13±5.14a | 32.09a | 46.89a | 7.81a |
|  | OE94 | 139.53±3.73a | 29.61b | 41.31b | 6.89a |

Each pot contained 10 kg dry soil. The soil was a sandy loamy with organic matter content of 13.61 g kg^-1^, total N of 0.69 g kg^-1^, alkaline hydrolysis N of 50.28 mg kg^-1^, available P of 17.73 mg kg^-1^, available K of 95.36 mg kg^-1^ and pH value of 8.24. and Ten seeds were sowed in each pot, and the plants were thinned to six plants per pot at three-leaf stage. The experiment had two topdressing treatments each with four replications. The ^15^N-labeled urea used in this experiment was provided by Shanghai Research Institute of Chemical Industry (Shanghai, China) with 46% N content, and 10.15% of ^15^N abundance ratio. The non-labeled N fertilizer was urea (46% N). The basal-N (72 mg N kg^-1^ soil) application with non-labeled N was mixed into the soil before seeding and the two topdress-N (48 mg N kg^-1^ soil) application with labeled N treatments was spread before irrigation at stem elongation and anthesis stage. A basal application of 32 mg P Kg^-1^ soil as calcium hydrogen phosphate and 62 mg K kg^-1^ soil as potassium chloride was applied to all treatments.

Measurements included grain and aboveground biomass yield of wheat, and the total N concentration in the plants and the soil profiles. Grain yield and crop residues were determined by harvesting all plants in each pot. ^15^N natural abundance of the soil were measured before the experiment. The natural ^15^N abundance of the soil was 0.365%. Total N and ^15^N abundance of samples were determined by the Kjeldahl method and by isotope-ratio analysis on a mass spectrometer, respectively, using the methods described by Buresh et al. (1982) and Keeney and Bremner (1967). All chemical analyses were performed in triplicate. Total plant N derived from labled fertilizer (Ndff) and from soil (Ndfs) was calculated by the isotope method of Hauck and Bremner (1976) as follows:

Grain Ndff = Grain ^15^N atom percent excess / urea^15^N atom percent excess×100 (1) Straw Ndff = Straw ^15^N atom percent excess / urea ^15^N atom percent excess×100 (2) Plant Ndff = (Grain Ndff × Grain N uptake + Straw Ndff × Straw N uptake) / Grain N uptake + Straw N uptake) × 100 (3)

**Table S2.** **The amino acids content in grains of the transgenic lines (OE53, OE57 and OE94) and wild type Ji5265 (WT)** . The data are presented as % of dry matter.

|  | High N | | | | Low N | | | |
| --- | --- | --- | --- | --- | --- | --- | --- | --- |
| Amino acid | WT | OE53 | OE57 | OE94 | WT | OE53 | OE57 | OE94 |
| Asp | 0.629 | 0.774** | 0.835** | 0.850** | 0.618 | 0.644 | 0.597 | 0.624 |
| Thr | 0.352 | 0.456** | 0.406* | 0.431** | 0.331 | 0.371 | 0.357 | 0.381 |
| Ser | 0.523 | 0.628** | 0.582* | 0.648** | 0.471 | 0.480 | 0.506 | 0.496 |
| Glu | 3.637 | 4.237** | 4.460** | 4.353** | 3.264 | 3.556 | 3.784 | 3.723 |
| Pro | 1.759 | 1.926** | 2.209** | 1.924** | 1.380 | 1.510* | 1.640** | 1.592* |
| Gly | 0.550 | 0.572 | 0.612 | 0.634 | 0.495 | 0.541 | 0.552 | 0.532 |
| Ala | 0.545 | 0.494 | 0.563 | 0.533 | 0.425 | 0.501 | 0.524 | 0.431 |
| Val | 0.390 | 0.424 | 0.415 | 0.450 | 0.412 | 0.468 | 0.478 | 0.443 |
| Ile | 0.418 | 0.418 | 0.433 | 0.505 | 0.427 | 0.444 | 0.457 | 0.469 |
| Leu | 0.789 | 0.822 | 0.805 | 0.825 | 0.758 | 0.842 | 0.837 | 0.922 |
| Tyr | 0.321 | 0.375 | 0.355 | 0.387 | 0.298 | 0.346 | 0.342 | 0.289 |
| Phe | 0.537 | 0.586 | 0.562 | 0.545 | 0.527 | 0.552 | 0.579 | 0.580 |
| His | 0.305 | 0.336 | 0.335 | 0.308 | 0.270 | 0.299 | 0.321 | 0.303 |
| Lys | 0.351 | 0.374 | 0.383 | 0.360 | 0.336 | 0.373 | 0.362 | 0.340 |
| Arg | 0.575 | 0.637 | 0.622 | 0.649 | 0.580 | 0.631 | 0.638 | 0.605 |
| Cys | 0.063 | 0.034 | 0.077 | 0.104 | 0.037 | 0.046 | 0.042 | 0.034 |
| Met | 0.163 | 0.218 | 0.182 | 0.198 | 0.196 | 0.186 | 0.178 | 0.188 |
| Total | 11.906 | 13.311 | 13.836 | 13.703 | 10.823 | 11.789 | 12.195 | 11.952 |

Data are means ± SE of four replicates. Asterisks indicate that the difference between the means of the transgenic lines and wild type was significant at the *P* < 0.05 (*) and *P* < 0.01 (**) level.

**Reference**

**Keeney DR, Bremner JM** (1967) Determination and isotope ratio analysis of different forms of nitrogen in soils. I. Urea. Soil Sci Soc Am Proc **31**:317–321

**Buresh RJ, Austin ER, Craswell ET** (1982) Analytical methods in N15 research. Fert Res **3**:37–62

**Hauck RD, Bremner JM** (1976) Use of tracers for soil and fertilizer nitrogen research. Adv Agron **28**:219–266
